# Exploring housing trajectories in later life and their links to demographic, socioeconomic and health characteristics: the register RELOC-AGE study

**DOI:** 10.1186/s12889-025-25920-1

**Published:** 2025-12-17

**Authors:** R. Samu Mtutu, Susanne Iwarsson, Jonas Björk, Nick Christie, Giedre Gefenaite

**Affiliations:** 1 Department of Health Sciences, Faculty of Medicine, Lund University, Lund, Sweden; 2 Department of Laboratory Medicine, Faculty of Medicine, Lund University, Lund, Sweden; 3 Clinical Studies Sweden, Forum South, Skåne University Hospital, Lund, Sweden

**Keywords:** Housing transitions, Ageing, Housing tenure

## Abstract

**Introduction:**

In Sweden, most older adults continue to age in dwellings they have lived in for many years, with a small proportion relocating. Longitudinal studies examining relocation histories, especially among younger old adults and those beyond frail populations, are scarce. This study aimed to describe individuals who stayed in their homes (stayers) and those who relocated (movers) while identifying and describing the housing trajectories of the movers and how they were predicted by (recent changes in) civil status, children in the household and health characteristics in the Swedish population.

**Materials and methods:**

The study population consisted of 106,962 adults born in 1957 and residing in Sweden. Movers were defined as individuals who had relocated at least once during 2013–2020. Data on housing, demographic, socioeconomic and health characteristics came from Swedish population registers. Based on housing type and tenure, housing trajectories were mapped using sequence and cluster analysis. We assessed the associations between the stayers and different relocation trajectories of movers and baseline demographic, socioeconomic and health conditions with the chi-square test and multinomial logistic regression.

**Results:**

The majority of participants did not relocate (*N* = 80,836; 76%). Among the movers (*N* = 26,136; 24%), eight housing trajectories were identified; three with relocations within the same and five transitioning into different housing types and tenures. Housing trajectories were predicted by disposable income, education, municipality type, changes in civil status and housing composition, as well as physical and mental health.

**Conclusion:**

The current study adds understanding of relocation trajectories as younger-old adults approach later life. Future research should consider adopting a life course perspective and a longer follow-up period to examine housing histories within different cultural and temporal contexts.

**Supplementary Information:**

The online version contains supplementary material available at 10.1186/s12889-025-25920-1.

## Introduction

The proportion of older adults continues to grow in many societies [1], and as people age, they tend to spend more time at home [2]. The home becomes an important source for individual well-being and promotes health and active ageing [3]. In Sweden, most older adults age in place – they remain in dwellings that they have lived in for a long time. Mobility rates for younger old adults are as low as 6–7% [4], and little is known about how these individuals navigate housing as they approach later life.

The expectation in Sweden is that most adults will eventually transition into homeownership [5]. However, a segment of the population never achieves homeownership either by choice or due to financial constraints. Individuals who have a history of renting are more likely to relocate compared to those who own their homes [6]. Research has shown that relocations in later life are heterogeneous, with a proportion of older people likely to experience several housing changes along the life course [7]. Such experiences include moving into homes where one can live independently for a long period of time as they strive to age in place [8]. Moreover, relocations can occur around retirement when leaving working life [9, 10]. In very old age, relocations are often associated with serious health decline as well as changes in household composition, such as widowhood or children leaving home, resulting in living alone [11, 12]. Furthermore, relocation patterns differ among men and women. Previous studies from Canada and Switzerland showed that among individuals aged 60+, men were more likely to move than women [13, 14].

Relocating at an older age is complex, especially for individuals who have lived in their homes for a long time. The home ceases to be just a dwelling and has meanings attached to it [15]. Additionally, in countries that have high homeownership rates, like the United States [16] and Sweden [6, 17], the home represents a significant source of wealth. With older households often occupying more housing space than needed, relocation may free up housing for younger families [18]. Recent research has shown that in Sweden, physical downsizing is often triggered by a mental health shock or an event that affects activities of daily living [12]. Thus, relocation in older age can be beneficial for individuals by freeing up financial resources when leaving homeownership, as well as for society. When older adults relocate, they can be expected to downsize physically or financially [14], and previous research has noted that when older adults relocate, they often aim for an improvement in the dwelling [19].

Housing trajectories have been studied from a life course perspective [20]. In these studies, the authors postulated that decisions about housing occur as part of various life events, such as having children, moving for labour market purposes, a change in civil status, or retirement. In the United Kingdom, a survey study investigated how housing histories in younger years related to well-being later in life and found that downward housing trajectories were associated with lower later-life wellbeing [21]. A multi-country study involving Sweden, Denmark and the Netherlands assessed how the family influenced the decision to leave home ownership [22]. A qualitative study from Germany has also investigated how changes in civil status and retirement intersect with housing trajectories [23]. These findings suggest that the interplay between housing and life events along the process of ageing is essential and underscores the need for a broader context when examining housing trajectories.

While there is literature examining relocations in the later phases of the ageing process [13, 14], studies investigating housing transitions from an earlier phase of the ageing process are scarce. Furthermore, there is a notable lack of longitudinal studies examining the relocation process among older adults who are not part of frail populations. Studies that mapped out housing trajectories based on tenures focused on younger cohorts aged 19–50 years, that is, during their most mobile period of life [5, 21]. Studies that investigated relocation patterns of older adults have used survey data with small sample sizes [11], short follow-up time or cross-sectional designs [24], or only investigated a move out of homeownership [22].

Most prior research studying predictors of relocation aimed at estimating the probability of changing housing using logistic regression [12, 14, 24], while some studies have incorporated survival analysis to assess the timing of housing transitions [11]. To our knowledge, only a couple of studies used sequence analysis to explore relocation trajectories of younger adults [5, 21]. Longitudinal studies linking housing trajectories to health outcomes, particularly among younger old adults, remain limited, with the exception of Kamis, Xu [25]. Our study addresses these limitations by using both sequence analysis and multinomial logistic regression to examine housing trajectories and their associations with health, which remain underexplored, particularly among younger old adults.

We hypothesise that, depending on their initial housing situation, individuals are likely to follow different trajectories influenced by their demographic, socioeconomic and health characteristics. For example, those in single-family housing at the age of 55 may be more inclined to downsize to smaller homes or transition to multi-family housing during the follow-up. In contrast, those who have had smaller living spaces and higher incomes may prefer to upsize or improve their housing by transitioning to single-family housing or modern multi-family housing. We will identify nuanced housing trajectories that reflect the actual diversity of housing choices available to older adults in Sweden, representing a key contribution that would be obscured by simple owner-renter dichotomies.

This study aimed to describe stayers and movers, identify and describe the housing trajectories of the movers and how they are predicted by (recent changes in) demographic, socioeconomic and health characteristics in the Swedish population aged 55 years in 2012.

## Materials and methods

### Study context and design

This study is part of the RELOC-AGE research program [26], which focuses on housing choices and relocation as related to active and healthy ageing in the Swedish population aged 55+. The current study is a longitudinal register-based study of the 1957 birth cohort (*N* = 106,962) selected from the Register RELOC-AGE project (55 years old at the beginning of the study period in 2012). Through the linkage of multiple population-based registers, Register RELOC-AGE allows the examination of relocations across a sample of over three million adults aged 55 + in Sweden (for details, see Gefenaite et al., submitted). For a full list of registers used in Register RELOC-AGE, see Supplementary Table S1 in the online Supplementary materials.

### Study population and study period

We focused on the cohort of adults residing in Sweden and aged 55–63 years old during the study period (*N* = 106,962), who were identified from the Total Population Register (TPR) maintained by Statistics Sweden with complete housing information. Of those, *N* = 80,826 (76%) did not relocate (hereafter referred to as “stayers”) and *N* = 26,136 (24%) relocated at least once during the study period. By utilising relocation data, we measured the number of times an individual changed address during 2013 and 2020.

### Data on housing characteristics and relocation

We used housing characteristics data from the Swedish Real Estate Property and Apartment Registers (REPR and AR, respectively) maintained by Statistics Sweden. As the number of individuals moving to residential care facilities was not sufficiently captured in the REPR, we additionally acquired residential care facility data from the National Register of Care and Social Services for the Elderly and Persons with Impairments (NRCSS), maintained by the National Board of Health and Welfare. Moves are measured once a year, and the characteristics of interest were the type and tenure of housing of each individual, which we combined into eight housing states (see Table 1). Individuals had to have at least five consecutive years of housing information in Sweden to be included in the study. When housing information was recorded as missing in the REPR and AR, and the individual was deceased in that year, we used the death as recorded in the TPR to complete the housing history.

The combination of tenure and type (see Table 1) allowed us to distinguish between different forms of housing, relevant in the Swedish housing context. The Swedish market predominantly includes the following housing types and tenure combinations: (i) owned single-family detached or attached row housing, (ii) tenant-owned multi-family housing, (iii) multi-family housing rented from public (requiring some queue time) or private landlords, usually with a secure tenancy agreement that individuals can rent for an unlimited time. Multi-family housing refers to buildings with multiple apartments. Additionally, there are (iv) other types of housing (e.g., business premises) that may include housing for tenants and (v) residential care facilities provided by the municipality for older adults who can no longer live independently in ordinary housing [27]. Other types and tenures, as depicted in Table 1, are also possible and represented in our data. However, for example, we were not able to distinguish between public and private landlords.

The importance of distinguishing between owned, tenant-owned and rented single-family and multi-family housing is that both the housing maintenance responsibilities and individual physical capacity requirements differ by tenure and type combinations, especially as people age [12]. For example, tenant-ownership refers to the ownership of an apartment in multi-family housing through cooperative ownership [5], where the tenant is responsible for maintaining the apartment and the cooperative association maintains the multi-family housing building [28]. However, as some tenure-type combinations were too rare for meaningful analysis, we merged two less prevalent housing tenure-type combinations.

**Table 1:** Eight housing States based on combinations of housing type and tenure

Housing type	Housing tenure	Housing state, *N* = 8
Single-family housing	Owner-occupied	Owned single-family housing
	Tenant-owned	Tenant-owned single-family housing
	Rented	Rented single-family housing
Multi-family housing	Tenant-owned	Tenant-owned multi-family housing
	Rented	Rented multi-family housing
Other housing	Tenant-owned	Tenant-owned other housing
	Rented	Rented other housing
Special housing: residential care facility	Rented	Residential care facility

### Data on demographic, socioeconomic and health characteristics

Demographic and socioeconomic data were obtained from the Longitudinal Integrated Database for Health Insurance and Labour Market Studies (LISA) maintained by Statistics Sweden. Sex was measured as a dichotomous variable (man/woman). Education and disposable income were measured in 2012. Education was defined as the highest educational level attained (mandatory education (≤ 9 years)/secondary education (≤ 12 years)/tertiary education (> 12 years)). Disposable income was categorised into quintiles based on annual equivalised family income, adjusted for household size, expressed in hundreds of Swedish kronor (SEK).

Changes in civil status and presence of children were assessed during the five years preceding the baseline (2007–2011) and categorised into no recent changes (status maintained for five consecutive years from 2007 to 2011, referred to as “long-term” or “continuous”) or recent changes (status changed in 2011). Accordingly, civil status was categorised as long-term single, long-term married or partnered, recently single (i.e., through divorce or widowhood), or recently married or partnered. Presence of children in the household was assessed as continuous presence of children, continuous absence of children, recent children, or recent empty nest.

To define the type of municipality, we used the 2011 aggregation of the 290 Swedish municipalities, which were compiled by the Swedish Association of Local Authorities and Regions. Municipalities were divided into three categories: (A) metropolitan areas and nearby municipalities, including cities with at least 200,000 inhabitants in the largest urban area and municipalities where ≥ 40% commute to such cities; (B) large cities and surrounding areas, including municipalities with ≥ 50,000 inhabitants (≥ 40,000 in the largest urban area), and municipalities with higher or lower commuting rates to large cities; and (C) smaller towns and rural areas, including municipalities with 15,000–39,999 inhabitants in the largest urban area, those with ≥ 30% commuting to smaller towns or other municipalities, and rural municipalities with a tourism profile (see supplementary Table S7).

We obtained information on hospitalisations based on inpatient and specialised outpatient care between 2007 and 2011 due to physical, mental and cognitive health conditions from the National Patient Register (NPR), which is maintained by the National Board of Health and Welfare. Each health condition was based on the timing of the prespecified diagnosis record based on the 10th revision of the International Statistical Classification of Diseases and Related Health Problems (ICD-10) (see Supplementary Table S2). Each health condition was categorised as none (no record during 2007–2011), recent (the first record in 2011, but no record in the previous four years) and long-term (at least one record during 2007–2010). A physical health condition was indicated by at least one record for the following conditions: anaemia, asplenia, asthma, cancer, cardiovascular diseases, chronic liver disease, endocrine, immunodeficiency, lung disease, neuromuscular disorders, obesity, renal disease, rheumatologic diseases, or stroke. A mental health condition was indicated by at least one record for the following conditions: mood affective disorders, anxiety, behavioural syndromes and emotional disorders, schizophrenia, disorders of adult personality, pervasive and specific developmental disorders, mental disorders caused by known physiological conditions, mental and behavioural disorders due to psychoactive substance use or unspecified mental disorders. A cognitive health condition was indicated by at least one record for the following conditions: dementia or intellectual disabilities.

### Statistical analysis

To assess the distribution of demographic, socioeconomic and health characteristics between stayers and movers, chi-square tests were performed for the descriptive analysis.

We applied sequence analysis to study the timing, duration and order of housing trajectories. Sequence analysis is a method used in social sciences and life course research [29], and has grown to be used in other disciplines to study health trajectories [30, 31], economic outcomes related to jobs and family formation [32, 33], as well as housing [5, 34]. Sequence analysis was performed only on the subsample that relocated. For stayers, the sequence does not change; hence, sequence analysis was not possible.

In the first step, we used sequence analysis to study the relocation trajectories of the movers. Sequences were created based on individual housing histories, and the most common sequences were grouped using optimal matching by a dissimilarity matrix, calculating the cost of transforming one housing state sequence into another [35]. Cost here refers to how much computing effort is required to transform one sequence into another. We used optimal matching in the TraMineR package in R that offers a constant substitution cost of two and an insertion/deletion cost of one [36]. This implies that transitioning between housing states is considered equally costly (for details, see Studer and Ritschard) [37] as housing quality in Sweden is generally good in both the rental and ownership markets [5].

In the second step, we used cluster analysis to describe typical trajectories found in the data, grouping homogenous sequences into one cluster and those that were dissimilar into other clusters [32, 37]. The clustered state sequences represented the housing trajectories. The WeightedCluster package in R was used, applying hierarchical clustering that uses the Ward method, which also Partitions Around the Medoids (PAM) [37]. Examining PAM statistics allowed us to predetermine and test several clusters, guiding cluster selection while taking into account what would make theoretical sense (see Supplementary Table S3 on PAM statistics).

In the third step, we used multinomial logistic regression with the identified housing trajectories as the dependent variable to assess the effects of demographic, socioeconomic and health characteristics on the housing trajectories. We conducted separate analyses for individuals starting in different housing states: owned single-family housing, rented multi-family housing and tenant-owned multi-family housing. Stayers were used as the reference category in the multinomial logistic regression analyses. By comparing movers to stayers within the same baseline housing state, we could study how different housing trajectories were predicted by demographic, socioeconomic and health characteristics.

The analysis was conducted using Stata 18 (StataCorp LLC, College Station, Texas). Results were expressed as odds ratios (OR) and their 95% confidence intervals (CI). For all analyses, results with p-values ≤ 0.05 were considered statistically significant.

### Ethical considerations

The Swedish Ethical Review Authority approved the Register RELOC-AGE project and inherent studies (dnr. 2020 − 01369; 2021 − 01124; 2024–02460−02). Informed consent to participate was not needed as this was a population register-based study using pseudonymized data. Instead, for participants to be able to express their decision to be excluded from Register RELOC-AGE, an opt-out message is available through the Lund University Research Portal [38]. The data were stored, managed and analysed on a high-security platform for handling sensitive data (LUSEC) at the Faculty of Medicine, Lund University. We followed regulations in accordance with the General Data Protection Regulation (GDPR) and ethical principles outlined in the Helsinki Declaration for research involving humans.

## Results

### Descriptive characteristics of stayers and movers

**Fig. 1:**
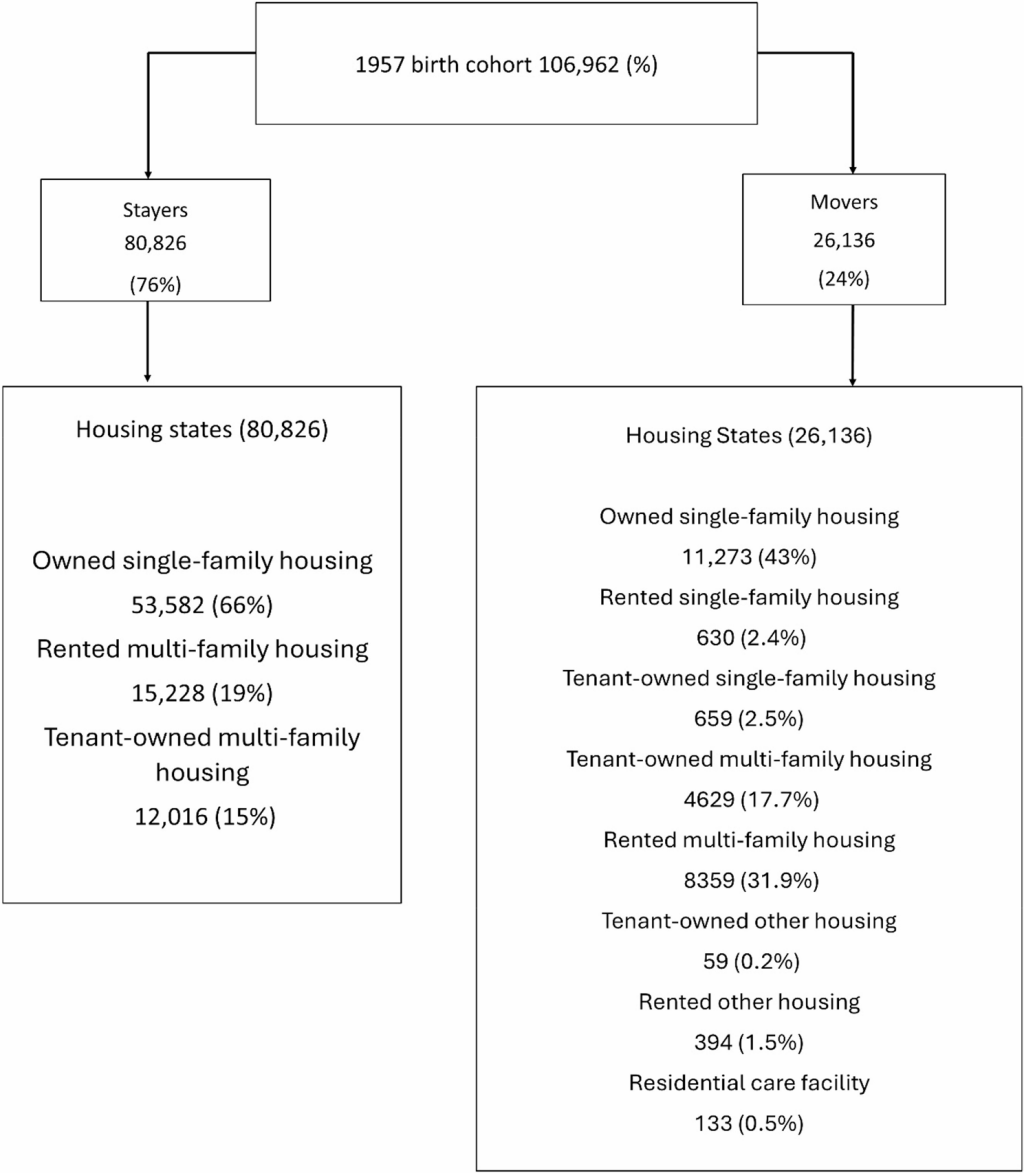
Flow chart of housing states at baseline for stayers and movers, *N* = 106,962

The majority of the stayers lived in owned single-family housing (66%), followed by participants in rented multi-family housing (19%) and tenant-owned multi-family housing (15%). Among movers, single-family housing (43%) was the most common, followed by rented multi-family housing (31%) and tenant-owned multi-family housing (17%) (Fig. 1).

There were more recently single individuals who were movers than stayers (22% vs. 11%, respectively). Almost half of movers and stayers had at least a secondary education, but there was a higher proportion of movers within the two lowest income quintiles than stayers (29% vs. 21%, respectively). There were slightly more women than men who moved (52%) (Table 2). Movers were slightly more likely to have a long-term mental health condition than stayers (10% vs. 7%, respectively).

**Table 2:** Descriptive characteristics of stayers and movers at baseline (*N* = 106,962)

	Stayers *N* = 80,826 (76%)	Movers *N* = 26,136 (24%)
Independent variable		
**Starting housing state**		
Owned single-family housing Rented single-family housing Tenant-owned single-family housing Tenant-owned multi-family housing Rented multi-family housing Tenant-owned other housing Rented other housing Residential care facility	53,582 (66.2%) - - 12,016 (15%) 15,228 (19%) - - -	11,273 (43%) 630 (2.4%) 659 (2.5%) 4629 (17.7%) 8359 (31.9%) 59 (0.2%) 394 (1.5%) 133 (0.5%)
**Sex**		
Women	40,048 (49%)	13,605 (52%)
**Education**		
Primary Secondary Tertiary	13,808 (17%) 39,122 (49%) 27,620 (34%)	4,577 (17%) 12,165 (47%) 9,266 (36%)
**Disposable income (quintiles of hundreds of SEK)**		
1 (0–1405) 2 (1405–1747) 3 (1747–2314) 4 (2314–3266,4) 5 (3266,4–434310)	9,348 (12%) 7,612 (9%) 16,233 (20%) 23,677 (29%) 23,956 (30%)	4,511 (17%) 3,036 (12%) 5,327 (20%) 6,307 (24%) 6,955 (27%)
**Civil status**		
Long-term partnered Long-term single Recently partnered Recently single	35,508 (44%) 24,653 (31%) 12,079 (15%) 8,586 (10%)	8,987 (34%) 8,457 (32%) 3,041 (12%) 5,651 (22%)
**Children in the home aged ≤ 17 years**		
Absence of children throughout Presence of children throughout Recently had kids Recent empty nest	10,827 (13%) 50,418 (63%) 2,579 (3%) 16,902 (21%)	3,358 (13%) 16,302 (63%) 858 (3%) 5,462 (21%)
**Municipal categories**		
Metropolitan areas and nearby municipalities Large cities and surrounding areas Smaller towns and rural areas	26,657 (33%) 32,191 (40%) 21,988 (27%)	9,514 (36%) 10,495 (40%) 6,127 (23%)
**Mental health condition**		
None Recent Long-term	74,806 (92.5%) 185 (0.2%) 5,835 (7.2%)	26,413 (89.5%) 79 (0.3%) 2,644 (10.1%)
**Physical health condition**		
None Recent Long-term	75,488 (93.3%) 245 (0.3%) 5,133 (6,4%)	24.089 (92.2%) 113 (0.4%) 1,934 (7.4%)
**Cognitive health condition**		
None Recent Long-term	80,746 (99.9%) 21 (0.03%) 59 (0.07%)	26,096 (99.9%) 9 (0.03%) 31 (0.12%)

Note: Stayers and movers were compared using a chi-square test at baseline in 2012

Housing trajectories of the movers

Housing trajectories of the movers

**Fig. 2:**
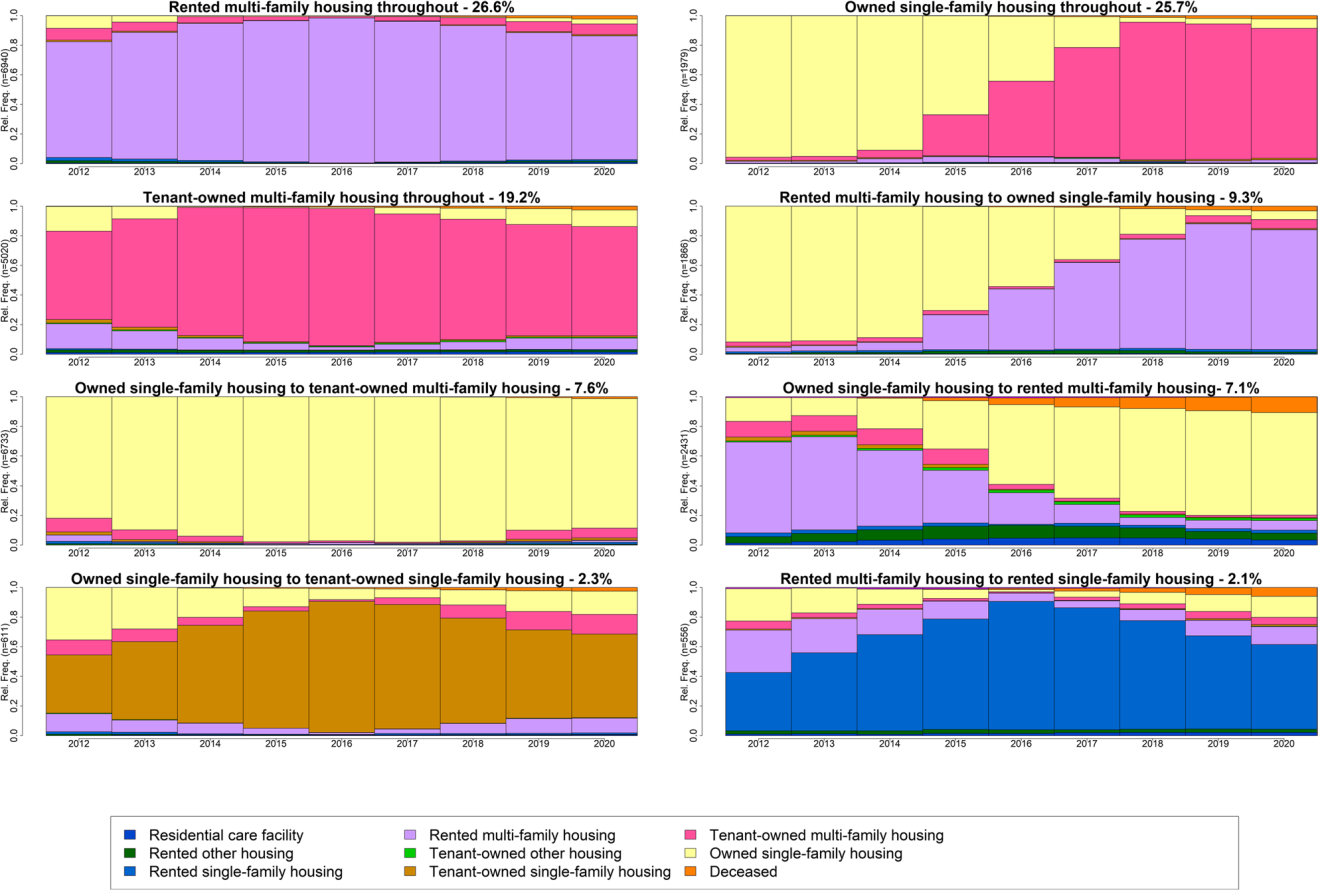
State distribution plots of movers into different housing state

The results of sequence and cluster analysis for movers are shown in Fig. 2 as state distribution plots derived from the cluster analysis for an eight-cluster solution, summarising sequences that are common based on the computed similarities. The eight clusters represent the housing trajectories. Participants who moved but remained within the same housing state spent an average of nine years. The most common housing trajectory at baseline among those who subsequently moved and remained within the same type and tenure was rented multi-family housing (*N* = 6,941), followed by owned single-family housing (*N* = 6,733) and tenant-owned multi-family housing (*N* = 5,020). For movers who changed type or tenure, we observed varying durations and timings of the moves across the trajectories followed. The most common trajectory involved participants spending an average of four years in rented multi-family housing and for the remainder of the study period transitioning into owned single-family housing (*N* = 2,430). The downsizing trajectories included transitions from owned single-family housing to either tenant-owned multi-family housing (*N* = 1,979), rented multi-family housing (*N* = 1,866) or tenant-owned single-family housing (*N* = 611). The least common trajectory was transitioning from an average of two years of rented multi-family housing to rented single-family housing (*N* = 556) for the remainder of the study period.

### Predictors of housing trajectories

#### Owned single-family housing at baseline

In the multinomial logistic regression analysis, the majority of the independent variables (i.e., demographic, socioeconomic and health-related factors) predicted transitions into different trajectories among those with a baseline of owned single-family housing. Among individuals starting in owned single-family housing, four trajectories were identified (Table 3) with stayers as the reference group.

**Table 3:** Predictors of moving to different housing trajectories among those in owned single-family housing as reference at baseline (N=64,771)

Independent variable	Owned single-family housing throughout	Owned single-family housing to tenant-owned multi-family housing	Owned single-family housing to rented multi-family housing	Owned single-family housing to tenant-owned single-family housing
	OR (95% CI)	OR (95% CI)	OR (95% CI)	OR (95% CI)
**Sex**				
Reference: Men				
Women	1.00 (0.94–1.05)	**1.10 (1.00–1.20)**	**1.41 (1.28–1.56)**	1.13 (0.96–1.33)
**Education**				
Reference: Primary				
Secondary	1.05 (0.97–1.14)	**1.27 (1.09–1.49)**	**0.84 (0.74–0.96)**	0.90 (0.71–1.14)
Tertiary	**1.14 (1.05–1.24)**	**1.70 (1.44–2.00)**	**0.77 (0.67–0.89)**	1.00 (0.78–1.29)
**Disposable income (quintiles of hundreds of SEK)**				
Reference: 1 (0–1405)				
2 (1405-1747)	**0.81 (0.72–0.91)**	1.09 (0.82–1.45)	**0.71 (0.58–0.85)**	0.97 (0.66–1.43)
3 (1747-2314)	**0.64 (0.57–0.70)**	1.14 (0.89–1.45)	**0.59 (0.50–0.69)**	0.81 (0.58–1.13)
4 (2314-3266,4)	**0.56 (0.51–0.62)**	**1.28 (1.02–1.61)**	**0.50 (0.43–0.59)**	0.78 (0.56–1.07)
5 (3266,4–434310)	**0.65 (0.59–0.72)**	**1.77 (1.41–2.22)**	**0.42 (0.36–0.50)**	0.89 (0.64–1.23)
**Civil status**				
Reference: Long-term partnered (2007-2011)				
Long-term single (2007-2011)	**1.30 (1.22–1.39) **	**1.41 (1.25–1.60) **	**2.07 (1.83–2.34)**	**1.72 (1.40–2.12)**
Recently partnered (2011)	1.03 (0.95–1.15)	0.95 (0.83–1.09)	1.10 (0.93–1.29)	1.06 (0.82–1.37**)**
Recently single (2011)	**2.35 (2.17–2.55)**	**3.78 (3.33–4.30)**	**5.25 (4.60–5.98)**	**3.70 (2.93–4.65)**
**Children in the home aged ≤17 years**				
Reference: Absence of children throughout (2007-2011)				
Presence of children throughout (2007-2011)	0.92 (0.85–1.00)	**1.19 (1.04–1.37)**	1.09 (0.95–1.26)	0.85 (0.66–1.10)
Recently had kids (2011)	0.88 (0.76–1.02)	**1.37 (1.09–1.72)**	1.04 (0.79–1.36)	1.40 (0.95–2.05)
Recent empty nest (2011)	0.94 (0.89–1.01)	**1.15 (1.03–1.28)**	1.05 (0.93–1.18)	0.94 (0.77–1.15)
**Municipality type**				
Reference: Metropolitan areas and nearby municipalities				
Large cities and surrounding areas	**0.83 (0.78–0.89)**	**0.60 (0.54–0.66)**	**1.16 (1.02–1.33)**	**0.78 (0.65–0.95)**
Smaller towns and rural areas	**0.82 (0.76–0.88)**	**0.40 (0.35–0.45)**	**1.15 (1.00–1.32)**	**0.49 (0.39–0.61)**
**Mental health condition**				
Reference: None (2007-2011)				
Long-term (2007-2011)	**1.19 (1.07–1.33)**	1.04 (0.84–1.29)	**1.62 (1.37–1.91)**	1.24 (0.88–1.73)
Recent (2011)	0.94 (0.52–1.72)	1.06 (0.37–2.97)	0.69 (0.24–1.95)	*
**Physical health condition**				
Reference: None (2007-2011)				
Long-term (2007-2011)	**1.13 (1.01–1.26)**	**1.27 (1.05–1.54)**	**1.45 (1.22–1.73)**	**1.43 (1.05–1.95)**
Recent (2011)	0.97 (0.54–1.76)	*	1.05 (0.44–2.47)	*
**Cognitive health condition**				
Reference: None (2007-2011)				
Long-term (2007-2011)	0.19 (0.26–1.43)	*	1.68 (0.56–5.06)	*
Recent (2011)	**4.75 (1.30–17.35)**	*	*	*
Constant	0.17 (0.15–0.20)	0.02 (0.01–0.03)	0.02 (0.02–0.03)	0.01 (0.01–0.02)
Observations	6.733	1.979	1.866	611

Women had 41% higher odds of transitioning to rented multi-family housing than men during the study period. Education higher than primary was associated with higher odds of transitioning to tenant-owned multi-family housing, but lower odds of transitioning to rented multi-family housing (Table 3). Higher income was generally associated with lower odds of moving from owned single-family housing to other housing trajectories, except for those in the two highest income quintiles, who were more likely to transition to tenant-owned multi-family housing.

Compared to long-term partnered individuals, long-term single as well as recently single were associated with changing the housing state and so transitioning from owned single-family housing into other forms of housing. As compared to no record of children during the five years preceding the study period, the presence of children since recently or throughout, as well as a recent empty nest, were associated with higher odds of transitioning to tenant-owned multi-family housing. Compared to residents of metropolitan areas, those living in large cities and surrounding areas were less likely to leave owned single-family housing for most trajectories, with the exception of transitions to rented multi-family housing (OR 1.16, 95% CI 1.02–1.33). Similarly, residents of smaller towns and rural areas showed reduced odds of most housing transitions, though they too were more likely than metropolitan residents to transition to rented multi-family housing (OR 1.15, 95% CI 1.00–1.32.00.32).

Health conditions predicted housing transitions as well; long-term mental health issues (OR 1.62, 95% CI 1.37–1.91) and long-term physical health issues (OR 1.45, 95% CI 1.22–1.73) had the strongest associations with transitioning from the owned single-family housing to rented multi-family housing. On the other hand, recent cognitive health condition (OR 4.75, 95% CI 1.30–17.35.30.35) was associated with a transition to another single-family housing. For further details and descriptive characteristics, see Supplementary Table S4.

#### Rented multi-family housing at baseline

Among those in rented multi-family housing at baseline, three trajectories were identified (Table 4) with stayers as the reference group. Women had lower odds of transitioning from rented multi-family housing to owned single-family housing (OR 0.89, 95% CI 0.81–0.97) and had higher odds of relocating to another rented multi-family housing. Individuals with higher than primary education were more likely to transition to owned single-family housing. Likewise, higher disposable income (income quintiles 3–5) was also associated with increased odds of moving to the owned single-family housing trajectory.

**Table 4:** Predictors of moving to different housing trajectories among those in rented multi-family housing at baseline (N=25,155)

Independent variables	Rented multi-family housing throughout	Rented multi-family housing to owned single-family housing	Rented multi-family housing to rented single-family housing
	OR (95% CI)	OR (95% CI)	OR (95% CI)
**Sex**			
Reference: Men			
Women	**1.06 (1.00–1.12)**	**0.89 (0.81–0.97)**	1.03 (0.86–1.22)
**Education**			
Reference: Primary			
Secondary	1.01 (0.94–1.09)	**1.17 (1.04–1.32)**	0.81 (0.66–1.00)
Tertiary	**1.12 (1.02–1.21)**	**1.32 (1.15–1.51)**	**0.77 (0.60–0.99)**
**Disposable income in (quintiles of hundreds of SEK)**			
Reference: 1 (0–1405)			
2 (1405-1747)	1.04 (0.95–1.14)	0.90 (0.77–1.05)	1.29 (0.98–1.68)
3 (1747-2314)	1.01 (0.93–1.09)	**1.18 (1.03–1.35)**	**1.40 (1.09–1.81)**
4 (2314-3266,4)	**1.12 (1.02–1.22)**	**1.46 (1.27–1.68)**	1.30 (0.98–1.72)
5 (3266,4–434310)	**1.12 (1.01–1.25)**	**2.59 (2.22–3.01)**	**2.00 (1.45–2.76)**
**Civil status**			
Reference: Long-term partnered (2007-2011)			
Long-term single (2007-2011)	**0.83 (0.77–0.90)**	**0.64 (0.57–0.73)**	**0.74 (0.59–0.92)**
Recently partnered (2011)	0.94 (0.84–1.05)	0.93 (0.79–1.09)	0.94 (0.68–1.29)
Recently single (2011)	**1.35 (1.24–1.48)**	1.06 (0.93–1.20)	0.98 (0.76–1.26)
**Children in the home aged ≤17 years**			
Reference: Absence of children (2007-2011)			
Presence of children (2007-2011)	**1.21 (1.10–1.33)**	1.09 (0.94–1.27)	**1.67 (1.28–2.16)**
Recently had kids (2011)	**1.60 (1.34–1.90)**	1.18 (0.88–1.59)	**2.24 (1.40–3.56)**
Recent empty nest (2011)	**1.37 (1.26–1.48)**	**1.41 (1.26–1.59)**	**1.58 (1.26–1.99)**
**Municipality type**			
Reference: Metropolitan areas and nearby municipalities			
Large cities and surrounding areas	**1.44 (1.35–1.54)**	**2.20 (1.98–2.44)**	**3.30 (2.63–4.15)**
Smaller towns and rural areas	**1.46 (1.35–1.59)**	**3.40 (3.02–3.84)**	**5.59 (4.38–7.15)**
**Mental health condition**			
Reference: None (2007-2011)			
Long-term (2007-2011)	1.06 (0.97–1.16)	1.14 (0.99–1.31)	0.96 (0.73–1.26)
Recent (2011)	0.98 (0.62–1.53)	1.50 (0.79–2.85)	0.89 (0.21–3.77)
**Physical health condition**			
Reference: None (2007-2011)			
Long-term (2007-2011)	0.96 (0.87–1.07)	0.95 (0.81–1.12)	1.09 (0.81–1.47)
Recent (2011)	1.00 (0.70–1.43)	1.13 (0.66–1.94)	1.57 (0.61–4.02)
**Cognitive health condition**			
Reference: None (2007-2011)			
Long-term (2007-2011)	1.23 (0.60–2.52)	2.14 (0.88–5.21)	1.15 (0.15–8.85)
Recent (2011)	0.30 (0.06–1.39)	0.79 (0.16–3.70)	*
Constant	0.28 (0.25–0.33)	0.08 (0.06–0.09)	0.01 (0.01–0.02)
Observations	6.941	2.430	556

Note: Multinomial logistic regression with stayers in rented multi-family housing (N = 12,016) as reference. Statistically significant results (*p*-value < 0.05) are bolded OR Odds ratio, CI Confidence interval* Could not be estimated due to a low number of observations

Individuals who were long-term single were less likely to transition to any trajectories of movers, whereas those who were recently single had higher odds of transitioning to another rented multi-family housing (OR 1.35, 95% CI 1.24–1.48). Those who recently had children, and who had children throughout the five years preceding the study period, had higher odds of transitioning to rented single- or multi-family housing. In contrast, recent empty nesters were more likely to transition to owned single-family housing.

Compared with individuals living in metropolitan areas, those living in large cities and surrounding municipalities had higher odds of transitioning to owned single-family housing (OR 2.20, 95% CI 1.98–2.44) and rented single-family housing trajectories (OR 3.30, 95% CI 2.63–4.15), with even stronger effects observed for those in smaller towns and rural areas (Table 4). None of the health variables were statistically significant predictors of housing transitions among individuals who were in rented multi-family housing at baseline. For further descriptive characteristics, see Supplementary Table S5.

#### Tenant-owned multi-family housing at baseline

Among individuals in tenant-owned multi-family housing at baseline, only one trajectory was identified: relocation to another tenant-owned multi-family dwelling. Therefore, the predictors presented in Table 5 effectively represent predictors of relocation within this housing type and tenure rather than transitions between different housing categories, with stayers as the reference group.

Tertiary education was associated with lower odds of relocating to another tenant-owned multi-family housing (OR 0.19, 95% CI 0.80–1.00.80.00). Moderate disposable income levels (quintiles 2–4) were associated with 12–31% higher odds of relocation (Table 5) compared to the lowest quintile.

Being recently single was associated with reduced odds of relocation, whereas both recently partnered and long-term single individuals had increased odds of transitioning to another tenant-owned multi-family housing. Individuals who had children consistently throughout the five years preceding the study period, those who recently had children, and recent empty nesters were all less likely to transition to another tenant-owned multi-family dwelling compared with those who never had children.

Living in large cities and surrounding areas, as well as smaller towns and rural areas, showed lower odds of relocation to another tenant-owned multi-family housing. Health conditions were not statistically significant predictors of relocation in this group. For further descriptive characteristics, see Supplementary Table S6.

**Table 5:** Predictors of moving to different housing trajectories among those in tenant-owned multi-family housing at baseline (*N* = 17,036)

Independent variable	Tenant-owned multi-family housing throughout
	OR (CI)
**Sex**	
Reference: Men	
Women	0.96 (0.90–1.03)
**Education**	
**Reference: Primary**	
Secondary	1.00 (0.90–1.12)
Tertiary	**0.19 (0.80–1.00** **)**
**Disposable income (quintiles of hundreds of SEK)**	
** Reference: 1 (0–1405)**	
2 (1405–1747) 3 (1747–2314) 4 (2314–3266,4) 5 (3266,4–434310)	**1.31 (1.12–1.53)** **1.20 (1.06–1.37)** **1.12 (1.00–1.27)** **0.82 (0.73–0.93)**
**Civil status**	
Reference: Long-term partnered	
Long-term single Recently partnered Recently single	**1.26 (1.16–1.37)** **1.23 (1.10–1.39)** **0.66 (0.60–0.73)**
**Children in the home aged ≤ 17 years**	
** Reference: Absence of children (2007–2011)**	
Presence of children throughout (2007–2011) Recently had kids (2011) Recent empty nest (2011)	**0.73 (0.65–0.82)** **0.67 (0.54–0.83)** **0.71 (0.65–0.77)**
**Municipality type**	
** Reference: Metropolitan areas and nearby municipalities**	
Large cities and surrounding areas Smaller towns and rural areas	**0.92 (0.85–0.99)** **0.84 (0.76–0.94)**
**Mental health condition**	
** Reference: None (2007–2011)**	
Long-term (2007–2011) Recent (2011)	0.92 (0.81–1.03) 0.72 (0.36–1.46)
**Physical health condition**	
**Reference: None (2007–2011)**	
Long-term (2007–2011) Recent (2011)	0.97 (0.84–1.12) 1.03 (0.59–1.79)
**Cognitive health condition**	
** Reference: None (2007–2011)**	
Long-term (2007–2011) Recent (2011)	0.54 (0.17–1.68) *
Constant	(2.90 (2.42–3.47)
Observations	17,036

Note: Multinomial logistic regression with stayers in tenant-owned multi-family housing (N = 12,016) as reference

Statistically significant results (*p*-value < 0.05) are bolded

*OR *Odds ratio, *CI *Confidence interval

*Could not be estimated due to a low number of observations

## Discussion

In this study, we examined the housing patterns of the 1957 birth cohort in Sweden over nine years (2012–2020), and their determinants, not the least studying how recent changes in life events predicted housing trajectories. The study thereby addresses a significant gap in the literature, as longitudinal register-based studies of housing mobility and its predictors among younger-old adults nearing retirement remain scarce. At baseline, most individuals lived in owned single-family housing: two-thirds among the stayers (66%) and less than half among the movers (43%). We identified eight housing trajectories among the movers, with most transitioning to tenant-owned or rented multi-family housing. Housing trajectories were predicted by diverse factors, including disposable income, education, changes in civil status and housing composition, municipality type and health conditions, with varying patterns by baseline housing tenure type.

As expected, most of the participants did not relocate during the study period, likely reflecting satisfaction with their circumstances, such as a stable housing composition or economic conditions. This finding is in line with a mixed-method study conducted in Germany showing that most pre-retirement older adults were satisfied with their current housing and felt attached to the neighbourhoods [39]. In our study, many relocated to new dwellings within the same housing state. While somewhat speculative, this finding suggests that these individuals had found housing tenure and type combinations that were both attractive and affordable for relocation.

To be able to study housing trajectories, we had to choose the number of clusters that best represented heterogeneity in the trajectories. As suggested by Liao et al. [35], a good typology should be “homogenous within types and very different between types”, which is illustrated in the state distribution plots (Fig. 2). We found that the optimal number of clusters in the current study could vary between six and eight. We ended up with a decision of eight, as using more clusters would have resulted in too low sample sizes for some of the clusters, thus making them less representative. Therefore, it should be noted that we only have one trajectory being compared in the tenant-owned multi-family housing trajectory (Table 5), as the cluster analysis did not produce a trajectory where individuals either moved to rented multi-family housing or owned single-family housing. This might be explained by the fact that the transition from tenant-owned multi-family housing to owned single-family housing is more visible in younger age groups in Sweden [5].

A previous study conducted in Sweden based on individuals aged 55 + who moved, found that as individuals age, their housing preferences change, with older adults who do move preferring other housing types than single-family housing [24]. The study also predicted a shortage of rented multi-family housing due to the preference for this housing option over homeownership. Our study adds to the previously mentioned study by showing that even in the early phases of ageing, housing choices and preferences are already apparent among those who relocate. This preference shift has practical implications. Recently, the National Board of Housing, Building and Planning [40] has forecasted housing needs for the ageing population that are not currently available in the existing housing stock. Additionally, forthcoming research from the Prospective RELOC-AGE suggests that those who were less satisfied with their housing were more likely to intend to move (Zingmark et al. forthcoming). Addressing this challenge will require rebuilding and constructing new housing that is tailored to the needs of older adults.

The 1957 cohort has also been suggested to have high mobility rates compared to older cohorts in a study with multiple European countries, which included Sweden [41]. The higher mobility rates may be linked to economic changes, such as better educational outcomes and job opportunities, which encouraged relocations for improved opportunities and, to an extent, access to different housing options compared to older cohorts. Our results partly reflect this, as higher disposable income and education were important predictors of the housing trajectory where individuals relocated from single-family housing to tenant-owned multi-family housing.

In our descriptive analysis, secondary education was the most common among both stayers and movers (almost 50%), with about one-third of the sample with a tertiary education. For older cohorts (those born in the 1940 s), it is likely that the most common educational level was lower than our cohort, as nine-year compulsory education was implemented between the 1960 s and early 70 s [42]. The 1940 s cohort was especially exposed to the Swedish “Million Program”, which aimed at building one million homes that were affordable and of better quality [19, 43]. Many in this cohort entered the housing market during the “Million Program” period, when homeownership was relatively more accessible. In contrast, our cohort (born in 1957) was met with a housing deregulation that led to increased housing costs for both owners and tenants, which affected people’s ability to enter the housing market [44]. This could have limited their ability to later relocate to different, that is, more expensive housing markets and meant that entering the owning housing market became more financially challenging for them [44].

Our findings showed that those with higher income and education were more likely to belong to the trajectory leading to tenant-owned housing. Because tenant-owned apartments require a mortgage, newly built tenant-owned multi-family housing is often expensive. Selecting tenant-owned multi-family housing may require a certain level of financial literacy, familiarity with mortgage processes, and social capital, making it more accessible to those with greater socioeconomic resources, such as people in later life who sell a single-family house, which they have owned for many years. Our findings suggest that tenant-ownership represents a viable long-term housing option for older adults, supporting both ageing in place and active ageing. This is particularly true for newly built apartments with elevators or located close to services that older adults find attractive [45]. In earlier phases of life, though, tenant-owned housing is often used as a stepping stone into the ownership housing market, especially for younger people and is particularly common in metropolitan areas in Sweden [5]. Housing planners should anticipate overlapping housing needs across age groups.

We found that among movers, individuals in single-family housing spent a mean of four years before relocating to another dwelling (Supplementary Figure S8). This suggests that relocation decisions may take time and require some reasoning, possibly including mixed feelings about the process [46]. Residential reasoning is not only related to the actual relocation but also includes what it entails to sell an estate, including the administrative tasks involved when the decision to sell is made. In contrast, those in the rental tenure appeared to have smoother transitions, as evidenced by individuals who spent a mean of two years in rented multi-family housing and transitioned to rented single-family housing for the remainder of the study period (Supplementary Figure S8). This suggests that residential reasoning is also shaped by tenure [46]. The finding that the timing of housing transitions among movers varies by tenure represents new knowledge and contributes to the existing literature in this field [47].

When it comes to civil status changes, we found that long-term and recently single individuals were more likely to leave owned single-family housing and transition into rented multi-dwelling housing. This pattern aligns with statistics and research showing that “grey divorces” are increasing [48]. Following a divorce, one household becomes two and at least one of the former partners typically must secure a new dwelling. This often results in renting, as this is less costly and more affordable than re-entering the ownership market when financial resources are separated [49]. Our results reflect this pattern, suggesting that economic constraints following partnership dissolution may play a significant role in shaping post-divorce housing transitions among individuals in this age group. Further research should continue exploring differences in relocation that are especially related to socioeconomic changes, as well as changes to one’s family status.

The findings of our results show clear gender differences in mobility. Women were more likely to be movers (Table 2), to be renters (e.g., see Tables 5 and 4) and to leave owned single-family housing — 55% of them transitioned into renting (Supplementary Table S4). The transition to rental housing likely reflects multiple factors, potentially including future financial and changing physical needs, and changes in civil status mentioned earlier. Evidence from a Dutch study indicates that women are at a greater risk of leaving homeownership compared to men, largely due to economic differences between men and women [49]. A qualitative Swedish study found that women with low pensions sometimes sold their homes, particularly after widowhood, and moved to tenant-owned or rented multi-family housing [50]. Our findings align with these patterns, though mobility-gender associations vary internationally; older men in Switzerland relocate more often than women due to higher incomes, and in Canada, widows show higher mobility rates [13, 14]. Avenues of future research should be to explore the long-term impact and interaction of civil status and gendered housing decisions in different cultural contexts.

Our sample consisted of younger old adults approaching later life. Health shocks are rather few, as indicated in our descriptive analysis, as comorbidities tend to increase with age [51], which is why we would expect their effect to become more pronounced as the population ages. A recent study in Sweden noted that a mental health shock or a health event that affects activities of daily living triggers relocation to another dwelling [12]. This was also shown in our study: having a mental health condition was a predictor of relocation, especially a transition from owned single-family housing to rented multi-family housing. Such a transition likely implies physical downsizing, which reduces household maintenance and may be better suited for individuals having mental health conditions. Potential confounding of union dissolution or job loss, which could be a common cause of both mental health and relocation and confound this relationship should be studied further.

We found that changes in household composition resulted in relocation, though the pattern differed by baseline tenure type. Among individuals starting in owned single-family housing, recently having children increased the odds of transitioning to tenant-owned multi-family housing, potentially signalling a downsize move or seeking more manageable housing. Similarly, among those in rented multi-family housing at baseline, having children in the household, whether recently or continuously present, predicted transition to rented single-family housing, likely reflecting the need for additional space. Previous research has noted that Sweden has relatively high mobility rates when children are younger, compared to other European countries like Spain and Austria [41]. In contrast, for those in tenant-owned multi-family housing at baseline, any child-related changes reduced the odds of relocating to another tenant-owned multi-family housing. Finally, economic constraints may limit housing mobility for families with young children, keeping them in the rental tenure when other tenure types may be preferred.

Unlike the study by Borg, Kawalerowicz [5], which focused on tenure trajectories followed by young adults, our study examined both tenure trajectories and within-tenure mobility for younger old individuals. This diversified perspective illustrates how different aspects of housing trajectories can be explored using similar data structures and analytical approaches. In our study, some individuals relocated to a new dwelling while still maintaining the same housing state (Fig. 2) during the study period. However, we could not determine whether these moves involved upsizing or downsizing, only that those individuals moved to another dwelling. Borg et al. [5] also found geographical links related to tenure trajectories, as certain trajectories are known to be concentrated in specific regions. While we did not focus on the geographical aspects of moving in our study, we know that, for instance, the trajectory of moving from rented multi-family housing to rented single-family housing identified in our study is more common in southern Sweden than in other parts of the country. This regional difference is likely due to the greater availability of rented single-family housing options in the southern parts of Sweden compared to elsewhere [52]. In comparison, some southern states in the United States, such as Florida, are quite popular retirement destinations for individuals approaching late life [9, 53]. Future studies could further investigate the mechanisms behind relocations to certain housing types and the geographic links in Sweden, as well as elsewhere. Residential mobility varied by municipality type. Individuals outside metropolitan areas were generally less likely to leave owned single-family housing, except for slightly higher odds of transitioning into rented multi-family housing. Conversely, among those in rented multi-family housing, living in large cities, surrounding areas, and especially small towns or rural municipalities was associated with transition into homeownership. As noted by an earlier study, this pattern likely reflects the lower cost and greater availability of single-family housing in rural and small town areas compared to metropolitan and surrounding areas [5]. Furthermore, this may indicate that those who make an upsize move at this stage in life have had smaller living spaces earlier in life and choose to transition to bigger and better housing as they age [14, 24, 54]. Thus, our results contribute to the literature showing that geographic differences in local housing markets are important predictors of relocation trajectories among the younger old adults.

### Strengths and limitations

One of the main strengths of our study was the use of a general population-based sample of younger old individuals, which is an understudied population segment when it comes to longitudinal studies on housing and relocation trajectories. The use of a population-based sample allowed us to provide insights that can be generalised to individuals who are approaching later life and navigating housing decisions.

We used methods that allowed us to map housing trajectories over time. While sequence analysis has been recently applied to health and residential disadvantages in general adult populations in the United States [25], to our knowledge, our study is the first to apply this analysis method to study housing and health links in Sweden. Given the differences in housing systems worldwide, studying housing trajectories of the younger old in Sweden adds to the current body of evidence on housing and health links in different contexts. Additionally, we presented a comprehensive sequence of housing histories allowing us to identify patterns that are seen in the younger old segment of the population, and related them to (recent changes in) demographic, socioeconomic and health characteristics, the latter still rather underexplored in relation to housing.

While the housing data in our study were very detailed, a nine-year period is relatively short to study relocation trajectories. A key strength of our descriptive approach was examining the predictive value of (changes in) demographic, socioeconomic and health characteristics on subsequent housing trajectories [55]. Future studies employing time-varying covariates could examine how life events dynamically interact with housing transitions over time to better understand causal mechanisms underlying the trajectories we identified. Additionally, since our analysis focuses on the 1957 cohort, the results should be generalised cautiously to other cohorts that encounter different housing policies and market changes.

We did not consider the geographical distances of relocation, which could have provided insight into whether younger old adults are relocating within the same municipalities or moving closer to family [56], or because of other reasons (Zingmark et al., forthcoming). Finally, due to data availability constraints when it comes to primary health care data in Sweden, we used diagnoses based on hospitalisations and specialised outpatient care to adjust for physical, mental and cognitive health conditions, which may underestimate health problems and health-related housing transitions.

## Conclusion

This study identified eight distinct housing trajectories among younger-old adults in Sweden, with most individuals remaining in the same housing throughout the study period. Regarding predictors of housing trajectories, multiple demographic, socio-economic and health-related factors emerged as significant. A consistent pattern across all baseline housing tenure types was that women had lower odds of transitioning to owned single-family housing compared to men. Socioeconomic factors also played key roles: higher education and income facilitated transitions to owned single-family housing and tenant-owned multi-family housing, while lower income was associated with remaining in or moving to rented multi-family housing. Recent life events, particularly a change in civil status, family composition changes, predicted specific trajectory patterns. Health conditions, particularly long-term mental and physical health, predicted moves from owned single-family housing to rented multi-family housing. The current study advances understanding of housing trajectories as younger old adults approach later life.

Future research could adopt a life course perspective with longer follow-up periods to examine how housing histories unfold across different cultural and temporal contexts, and investigate how these early-old-age housing transitions influence long-term housing stability and wellbeing in later life.

## Supplementary Information

Supplementary Material 1.

## Data Availability

Sharing individual-level data requires new ethical approval and a confidentiality review by Lund University.

## Abbreviations

AR: Apartment Register
CI: Confidence interval
LISA: Longitudinal Integrated Database for Health Insurance and Labour Market Studies
NRCSS: National Register of Care and Social Services for the Elderly and Persons with Impairments
OR: Odds ratio
PAM: Partitions Around the Medoids
REPR: Real Estate Property Register
SEK: Swedish Krona
TPR: Total Population Register

## References

[1] World Health Organization. Decade of healthy ageing: baseline report. World Health Organization; 2021.

[2] Kylén M, et al. Meaning of home and health dynamics among younger older people in Sweden. Eur J Ageing. 2019;16(3):305–15. 31543725 10.1007/s10433-019-00501-5 PMC6728404

[3] Mahler M, et al. Home as a health promotion setting for older adults. Scand J Public Health. 2014;42(15suppl):36–40. 25416572 10.1177/1403494814556648

[4] Sweden S. Statistics Sweden Moving within Sweden. 2023 [cited 2024 12 September]; Available from: https://www.statistikdatabasen.scb.se/pxweb/en/ssd/START__BE__BE0101__BE0101J/FlyttningarInrk/table/tableViewLayout1/

[5] Borg I, Kawalerowicz J, Andersson EK. Socio-spatial stratification of housing tenure trajectories in Sweden – A longitudinal cohort study. Adv Life Course Res. 2022;52:100467. 36652322 10.1016/j.alcr.2022.100467

[6] Fischer PA, Malmberg G. Settled people don’t move: on life course and (im-)mobility in Sweden. Int J Popul Geogr. 2001;7(5):357–71.

[7] Golant SM. The quest for residential normalcy by older adults: relocation but one pathway. J Aging Stud. 2011;25(3):193–205.

[8] Granbom M, et al. A public health perspective to environmental barriers and accessibility problems for senior citizens living in ordinary housing. BMC Public Health. 2016;16(1):772. 27514631 10.1186/s12889-016-3369-2 PMC4982418

[9] Litwak E, Longino CF Jr. Migration patterns among the elderly: a developmental perspective. Gerontologist. 1987;27(3):266–72. 3609792 10.1093/geront/27.3.266

[10] Eriksson E, et al. Perceived housing in relation to retirement and relocation: a qualitative interview study among older adults. Int J Environ Res Public Health. 2022. 10.3390/ijerph192013314. 36293895 10.3390/ijerph192013314 PMC9602647

[11] Painter G, Lee K. Housing tenure transitions of older households: life cycle, demographic, and familial factors. Reg Sci Urban Econ. 2009;39(6):749–60.

[12] Costa-Font J, Vilaplana-Prieto C. Health shocks and housing downsizing: how persistent is ‘ageing in place’? J Econ Behav Organ. 2022;204:490–508.

[13] Edmonston B, Lee SM. Residential mobility of elderly canadians: trends and determinants. Can J Aging / La Revue Canadienne Du Vieillissement. 2014;33(4):378–99. 10.1017/S071498081400035X 25298179

[14] Huggenberger Y, Wagner J, Wanzenried G. The determinants of the mobility patterns of the elderly in Switzerland. J Housing Built Environ. 2023;38(3):2151–84.

[15] Oswald F, Wahl H-W. Dimensions of the meaning of home in later life. In Rowles GH, Chaudry H. (Eds.), Home and identity in late life: International perspectives. New York: Springer; 2005:21–45.

[16] Venti SF, Wise DA. Aging and housing equity: Another look, in Perspectives on the economics of aging. NBER Working Paper No. w8608, University of Chicago Press; 2004. p. 127–80.

[17] Chiuri MC, Jappelli T. Do the elderly reduce housing equity? An international comparison. J Popul Econ. 2010;23(2):643–63.

[18] Clark WAV, Deurloo MC. Aging in place and housing over-consumption. J Housing Built Environ. 2006;21(3):257–70.

[19] Abramsson M, Andersson E. Changing residential mobility rates of older people in Sweden. Aging Soc. 2012;32(6):963–82.

[20] Pollock G. Holistic trajectories: a study of combined employment, housing and family careers by using multiple-sequence analysis. J Royal Stat Society: Ser (Statistics Society). 2007;170(1):167–83.

[21] Vanhoutte B, Wahrendorf M, Nazroo J. Duration, timing and order: how housing histories relate to later life wellbeing. Longitud Life Course Stud. 2017;8(3):227–44.

[22] Herbers DJ, Mulder CH, Mòdenes JA. Moving out of home ownership in later life: the influence of the family and housing careers. Hous Stud. 2014;29(7):910–36.

[23] Wanka A, et al. Moving in together in later life: making spaces into places as a joint endeavor. J Aging Stud. 2024;68:101191. 38458716 10.1016/j.jaging.2023.101191

[24] Abramsson M, Andersson E. Changing preferences with ageing – housing choices and housing plans of older people. Hous Theory Soc. 2016;33(2):217–41.

[25] Kamis C, et al. Linking sequences of exposure to residential (dis)advantage, individual socioeconomic status, and health. Health Place. 2024;88:103262. 38833849 10.1016/j.healthplace.2024.103262 PMC11878194

[26] Zingmark M, et al. Exploring associations of housing, relocation, and active and healthy aging in Sweden: protocol for a prospective longitudinal mixed methods study. JMIR Res Protoc. 2021;10(9):e31137. 34546172 10.2196/31137 PMC8493467

[27] Statistics Sweden S. Number of dwellings by type of building and year. 2021 [cited 2023; Available from: https://www.statistikdatabasen.scb.se/pxweb/en/ssd/START__BO__BO0104__BO0104D/BO0104T01/table/tableViewLayout1/

[28] Granath Hansson A, Ekbäck P, Paulsson J. The sliding scale between usufruct and ownership: the example of Swedish multi-family housing. Land. 2021;10(3):311.

[29] Abbott A. Sequence analysis: new methods for old ideas. Ann Rev Sociol. 1995;21:93–113.

[30] Vogt V, Scholz SM, Sundmacher L. Applying sequence clustering techniques to explore practice-based ambulatory care pathways in insurance claims data. Eur J Pub Health. 2018;28(2):214–9. 29040495 10.1093/eurpub/ckx169

[31] Elsenburg LK, et al. Application of life course trajectory methods to public health data: A comparison of sequence analysis and group-based multi-trajectory modeling for modelling childhood adversity trajectories. Soc Sci Med. 2024;340:116449. 38091856 10.1016/j.socscimed.2023.116449

[32] Ritschard G, Studer M. Sequence Analysis: Where Are We, Where Are We Going? in Sequence Analysis and Related Approaches: Innovative Methods and Applications, G. Ritschard and M. Studer, Editors. Springer International Publishing: Cham. 2018;10:1–11.

[33] Anyadike-Danes M, McVicar D. You’ll never walk alone: childhood influences and male career path clusters. Labour Econ. 2005;12(4):511–30.

[34] Nelson R, Warnier M, Verma T. Housing inequalities: the space-time geography of housing policies. Cities. 2024;145:104727.

[35] Liao TF, et al. Sequence analysis: its past, present, and future. Soc Sci Res. 2022;107:102772. 36058612 10.1016/j.ssresearch.2022.102772

[36] Gabadinho A, et al. Analyzing and visualizing state sequences in R with traminer. J Stat Softw. 2011;40(4):1–37.

[37] Studer M, Ritschard G. What matters in differences between life trajectories: a comparative review of sequence dissimilarity measures. J Royal Stat Society: Ser (Statistics Society). 2016;179(2):481–511.

[38] LU Research Portal. *RELOC-AGE: *How do housing choices and relocation matter for active and healthy ageing? 2021 25 June 2025]; Available from: https://portal.research.lu.se/en/projects/reloc-age-how-do-housing-choices-and-relocation-matter-for-active

[39] Kramer C, Pfaffenbach C. Should I stay or should I go? Housing preferences upon retirement in Germany. J Housing Built Environ. 2016;31(2):239–56.

[40] Boverket NBOH, Building and, Planning. Need for housing construction 2023–2030. (In Swedish). National Board Of Housing, Building and Planning; 2023.

[41] Bernard A, Vidal S. Does moving in childhood and adolescence affect residential mobility in adulthood? An analysis of long-term individual residential trajectories in 11 European countries. Popul Space Place. 2020;26(1):e2286.

[42] Hällsten M, Thaning M. Multiple dimensions of social background and horizontal educational attainment in Sweden. Res Social Stratif Mobil. 2018;56:40–52.

[43] Boverket NBOH. Building and planning Swedish policy for housing, planning and construction for 130 years. Swedish). National Board of Housing, Building and Planning; 2007.

[44] Welin L, Bildsten L. The housing market in Sweden: a political-historical perspective. 2017.

[45] Kylén M, et al. Housing attribute preferences when considering relocation in older age. Innov Aging. 2023;7(Supplement1):223–223.

[46] Granbom M. Relocation and residential reasoning in very old age. 2014, Doctoral Thesis, Lund University.

[47] Aarland K, Reid CK. Homeownership and residential stability: does tenure really make a difference? Int J Hous Policy. 2019;19(2):165–91.

[48] Kawalerowicz J et al. Splitting up late: housing changes around the time of divorce for older men and women in Sweden*.* 2024.

[49] Feijten P. Union dissolution, unemployment and moving out of homeownership. Eur Sociol Rev. 2005;21(1):59–71.

[50] Yadav A, Granbom M, Iwarsson S. Older women with a low pension, living in sweden: strategies to age in place and thoughts about future housing. Hous Soc. 2023;50(3):357–74.

[51] Divo MJ, Martinez CH, Mannino DM. Ageing and the epidemiology of multimorbidity. Eur Respir J. 2014;44(4):1055–68. 25142482 10.1183/09031936.00059814 PMC4918092

[52] Statistics Sweden S. Number of dwellings by region, type of building and tenure. Year 2012–2020. 2024 27 November 2024; Available from: https://www.statistikdatabasen.scb.se/pxweb/en/ssd/START__BO__BO0104__BO0104D/BO0104T04/

[53] Lovegreen LD, Kahana E, Kahana B. Residential relocation of amenity migrants to florida: unpacking post-amenity moves. J Aging Health. 2010;22(7):1001–28. 20647535 10.1177/0898264310374507

[54] Burgess G, Quinio V. Unpicking the downsizing discourse: understanding the housing moves made by older people in England. Hous Stud. 2021;36(8):1177–92.

[55] Judd B, et al. Downsizing amongst older Australians. Australian Housing and Urban Research Institute; 2012.

[56] Artamonova A, et al. Adult children’s gender, number and proximity and older parents’ moves to institutions: evidence from Sweden. Aging Soc. 2023;43(2):342–72.

