# Supplementary material for: Exploring housing trajectories in later life and their links to demographic, socioeconomic and health characteristics: the register RELOC-AGE study
Source: BMC Public Health. 2025 Dec 17;26:289. doi: 10.1186/s12889-025-25920-1 (PMC12828926; doi:10.1186/s12889-025-25920-1)
Supplement: Supplementary file 1 — Supplementary Material 1. [file 12889_2025_25920_MOESM1_ESM.docx]

**Exploring housing trajectories in later life and their links to demographic, socioeconomic and health characteristics: The Register RELOC-AGE study**

R. Samu Mtutu^1^, MPH

Susanne Iwarsson^1^, PhD

Jonas Björk^2,3^, PhD

Nick Christie^1^, PhD

Giedre Gefenaite^1^, PhD

1. Department of Health Sciences, Faculty of Medicine, Lund University, Lund, Sweden
2. Department of Laboratory Medicine, Faculty of Medicine, Lund University, Lund, Sweden
3. Clinical Studies Sweden, Forum South, Skåne University Hospital, Lund, Sweden

Corresponding author: R. Samu Mtutu [regina.mtutu@med.lu.se](mailto:regina.mtutu@med.lu.se)

**Supplementary Materials**

Supplementary Table S1 Registers included in Register RELOC-AGE

| **Register Name** | **Start year in Register RELOC-AGE** | **Last year in Register RELOC-AGE** |
| --- | --- | --- |
| Total Population Register (TPR) | 1987 | 2020 |
| Longitudinal integrated database for health insurance and labour market studies (LISA) | 1990 | 2020 |
| National Patient Register (NPR) | 1987 | 2021 |
| Cause of Death Register (CDR) | 1987 | 2020 |
| Drug Prescription Register (DPR) | 2005 | 2021 |
| Municipal Health Care Register (MHCR) | 2007 | 2021 |
| Swedish internet-based surveillance system for communicable diseases (SmiNet) | 2007 | 2021 |
| Interventions for Elderly and People with Disabilities Register (IEPDR) | 2007 | 2021 |
| Swedish Intensive Care Register (SIR) | 2007 | 2021 |
| Apartment Register (AR) | 2012 | 2021 |
| Real Estate Property Register (REPR) | 1987 | 2021 |
| Geographical database (GD) | 1987 | 2021 |
| Scout | 2008 | 2019 |

Supplementary Table S2 Health conditions and respective ICD-10 codes

| **Condition** | **ICD 10** |
| --- | --- |
| **Physical health**  Anaemia | D50-53, D55-64 |
| Asplenia | 738D, Q20-28, Z908 |
| Asthma | 493A-B and 49X |
| Cancer | C00-14, C30-39, C40-41, C43-97 |
| Cardiovascular disease | 093, 391, 401, 112W, I05-109, I11, I13, I20-52, I260, I 269, I398, I412, I790, I970, I971, Q874, R001, R011-012, T817, T828, |
| Chronic liver disease | 571, 571E, 571X, K70-77, K754, K769 |
| Dementia | 290,293,F01,F03,F05, G30, G30-, G300,  G301, G308, G309, G31,G91,G94 |
| Endocrine | 244,250,E10-14, E40-46, E89 |
| Immunodeficiency | B20, 279, D80-89, D898, Z21, Z94 |
| Lung disease | 490-496, 500-508, 510-519, 518W, 519X, 714W, J182, J40-44, J47, J60-70, J80-82, J84-86, J90-94, J96, J99, M051 |
| Neuromuscular disorders | 358, 359, 358A, 378H, G70-73 |
| Obesity | 278A, 278W, E660-662, E668, E669 |
| Renal disease | 274X, 592A, 593X, M103, N00-08, N10-200, N289 |
| Rheumatologic diseases | M08, M12, M30-M36, M350, M355, |
| Stroke | 348, 438, G93, I679, I679, I69, I690, I690A, I690B, I691, I693, I694 |
| **Mental health**  Mental disorders due to known physiological conditions | F00-F09 |
| Mental and behavioural disorders due to psychoactive substance use | F10-F19 F10-19, F171.1, F171.2 |
| Schizophrenia, schizotypal, delusional, and other non-mood psychotic disorders | F20-F29 |
| Mood affective disorders | F30, F31, F32, F33, F34, F38, F39 |
| Anxiety, dissociative, stress-related, somatoform and other nonpsychotic mental disorders | F40-F48 F400, F401, F402, F402G, F409-413, F418, F419, F419P, F421, F422, F428-F432, F4320, F4322, F438, F438A, F438W, F439, F440, F442, F444, F445, F447-F449, F450-454, F458, F459, F480, F488, F489 |
| Behavioural syndromes associated with physiological disturbances and physical factors | F50-F59 |
| Disorders of adult personality and behaviour | F60-F69 |
| Pervasive and specific developmental disorders | F80-F89 |
| Behavioural and emotional disorders with onset usually occurring in childhood and adolescence | F90-F98 |
| Unspecified mental disorder | F99-F99 |
| **Cognitive health**  Dementia | F01, F03 |
| Intellectual disabilities | F70-F79 |

Supplementary Table S3 PAM statistics

| **Clusters** | **PBC** | **HG** | **HGSD** | **ASW** | **ASWw** | **CH** | **R2** | **CHsq** | **R2sq** | **HC** |
| --- | --- | --- | --- | --- | --- | --- | --- | --- | --- | --- |
| Cluster4 | 0,759 | 0,922 | 0,907 | 0,507 | 0,507 | 10471,2 | 0,545 | 23697,08 | 0,731 | 0,059 |
| Cluster5 | 0,710 | 0,898 | 0,898 | 0,469 | 0,470 | 8962,506 | 0,470 | 19670,58 | 0,705 | 0,079 |
| Cluster6 | 0,687 | 0,881 | 0,874 | 0,461 | 0,461 | 7808,894 | 0,431 | 16938,32 | 0,742 | 0,078 |
| Cluster7 | 0,712 | 0,919 | 0,847 | 0,481 | 0,481 | 8145,167 | 0,447 | 16803,81 | 0,794 | 0,067 |
| Cluster8 | 0,719 | 0,948 | 0,905 | 0,507 | 0,507 | 6877,207 | 0,464 | 11712,66 | 0,684 | 0,046 |
| Cluster9 | 0,714 | 0,951 | 0,909 | 0,502 | 0,502 | 6615,634 | 0,466 | 11564,19 | 0,667 | 0,063 |
| Cluster10 | 0,709 | 0,954 | 0,947 | 0,498 | 0,498 | 6524,537 | 0,464 | 15989,05 | 0,846 | 0,065 |
| Cluster11 | 0,708 | 0,919 | 0,953 | 0,531 | 0,531 | 6478,527 | 0,504 | 15540,42 | 0,857 | 0,035 |
| Cluster12 | 0,682 | 0,923 | 0,916 | 0,506 | 0,506 | 5777,35 | 0,506 | 14658,85 | 0,846 | 0,083 |
| Cluster13 | 0,687 | 0,972 | 0,971 | 0,520 | 0,520 | 5802,106 | 0,506 | 14069,06 | 0,835 | 0,078 |
| Cluster14 | 0,690 | 0,951 | 0,959 | 0,531 | 0,531 | 5481,437 | 0,506 | 15379,45 | 0,932 | 0,063 |
| Cluster15 | 0,664 | 0,977 | 0,968 | 0,514 | 0,514 | 5384,665 | 0,506 | 15226,26 | 0,809 | 0,023 |

*Note: this table shows partitioning statistics, we are interested in the Point Biserial Correlation (PBC), Hubert’s C (HC), ASW and Hubert’s Gamma (HG) [48].*

**Supplementary Table S4: Descriptive characteristics of stayers and movers who lived in owned single-family housing at baseline (N=64,768)**.

|  | **Stayers** | **Movers** from owned single-family housing to N=11,186 (%) | | | |
| --- | --- | --- | --- | --- | --- |
| Independent variable | Owned single-family housing  N=53,582 (%) | Owned single-family housing N=6,733 (%) | Tenant-owned multi-family housing N=1,976 (%) | Tenant-owned single family housing  hip N=611 (%) | Rented multi-family housing  N=1,866 (%) |
| **Sex**  Women | 25,742 (48) | 3,232 (48) | 996 (50) | 307 (50) | 1,025 (55) |
| **Education**  Primary  Secondary  Tertiary | 8,443 (16)  26,139 (49)  18,900 (35) | 1,020 (15)  3,233 (48)  2,463 (37) | 200 (10)  822 (42)  957 (48) | 101 (17)  275 (45)  234 (38) | 372 (20)  943 (51)  546 (29) |
| **Disposable income in quintiles (hundreds of SEK)**  1 (0-1405)  2 (1405-1747)  3 (1747-2314)  4 (2314-3266,4)  5 (3266,4-434310) | 3,348 (6)  3,806 (7)  10,016 (19)  17,586 (33)  18,826 (35) | 707 (11)  631 (9)  1,254 (19)  1,848 (27)  2,293 (34) | 95 (5)  114 (6)  303 (15)  564 (28)  903 (46) | 56 (9)  55 (9)  114 (19)  174 (28)  212 (35) | 285 (15.3)  218 (12)  434 (23.3)  528 (28)  401 (21) |
| **Civil status**  Long-term partnered (2007-2011)  Long-term single (2007-2011)  Recently partnered (2011)  Recently single (2011) | 7,925 (15)  30,750 (57)  1,933 (4)  12,924 (24) | 2,971 (44)  1,749 (26)  1,006 (15)  1,007 (15) | 882 (45)  427 (22)  281 (14)  389 (19) | 580 (31)  596 (32)  209 (11)  481 (26) | 240 (39)  168 (28)  84 (14)  119 (19) |
| **Children in the home aged ≤17 years**  Absence of children (2007-2011)  Presence of children (2007-2010)  Recently had kids (2011)  Recent empty nest (2011) | 7,925 (15)  30,750 (57)  1,933 (3.6)  12,924 (24) | 956 (14)  4005 (59.7)  224 (3.3)  1529 (22.8) | 317 (16)  1042 (53)  89 (4.5)  526 (26) | 269 (14.5)  1121 (60)  62 (3.3)  410 (22) | 77 (13)  362 (59)  31 (5.1)  136 (22.4) |
| **Municipality type**  Metropolitan areas and nearby municipalities  Large cities and surrounding areas  Smaller towns and rural areas | 12,535 (23.4)  22,749 (42.5)  18,298 (34.2) | 1,796 (27)  2,731 (40)  2,204 (33) | 796 (40.2)  780 (39.4)  402 (20.3) | 351 (19)  813 (44)  702 (37) | 194 (32)  274 (45)  143 (23) |
| **Mental health condition**  None (2007-2011)  Long-term (2007-2010)  Recent (2011) | 51,034 (95)  2,457 (4.6)  91 (0.2) | 6,303 (94)  417 (6.2)  13 (0.2) | 1,883 (95)  92 (4.7)  4 (0.2) | 1,683 (90.2)  178 (9.5)  5 (0.3) | 573 (94)  38 (6.2)  0 (0) |
| **Physical health condition**  None (2007-2011)  Long-term (2007-2010)  Recent (2011) | 50,504 (94)  2,992 (5.6)  86 (0.2) | 6,293 (93)  426 (6.8)  14 (0.2) | 1,852 (94)  127 (6)  0 (0) | 1,702 (91)  158 (8.6)  6 (0.3) | 566 (93)  45 (7)  0 (0) |
| **Cognitive health condition**  None (2007-2011)  Long-term (2007-2010)  Recent (2011) | 53,546 (99.9)  30 (0.1)  6 (0.01) | 6,728 (99.9)  1 (0.01)  4 (0.1) | 1,979 (100)  0 (0)  0 (0) | 1,862 (99.8)  4 (0.2)  0 (0) | 611 (100)  0 (0)  0 (0) |

*Note: The stayers and movers are compared based on the same housing situation at baseline using a chi-square test. The table also shows where individuals moved to between 2012 and 2020.*

**Supplementary Table S5: Descriptive characteristics of stayers and movers who lived in multi-family housing renting at baseline, N=25,155.**

|  | **Stayers**  N=15,228 (%) | **Movers** N=9,927 (%) | | |
| --- | --- | --- | --- | --- |
|  | Rented multi-family housing | **Multi-family housing** to | | |
| Independent variable |  | Rented multi-family housing N=6,941 | Rented single-family housing  N=556 | Owned single-family housing  N=2,430 |
| **Sex**  Women | 7,901 (52) | 3,777 (54) | 292 (53) | 13,184 (52) |
| **Education**  Primary  Secondary  Tertiary | 3,701 (25)  7,518 (50)  3,878 (25) | 1,631 (23.6)  3,339 (49)  1,914 (27.8) | 155 (28)  269 (49)  130 (23) | 5,962 (24)  12,321 (49)  6,661 (26) |
| **Disposable income in quintiles (SEK)**  1 (0-1405)  2 (1405-1747)  3 (1747-2314)  4 (2314-3266,4)  5 (3266,4-434310) | 4,628 (30)  2,612 (17.2)  3,588 (23.7)  2,848 (18.7)  1,552 (10.2) | 2061 (30)  1210 (17)  1588 (23)  1351 (19.5)  731 (10.5) | 138 (25)  101 (18)  146 (26)  99 (18)  72 (13) | 7404 (29,4)  4224 (17)  5860 (23)  4817 (19.2)  2850 (11) |
| **Civil status**  Long-term partnered (2007-2011)  Long-term single (2007-2011)  Recently partnered (2011)  Recently single (2011 | 3,605 (24)  7,389 (49)  1,344 (9)  2,890 (18) | 1,734 (25)  2,781 (40)  598 (9)  1,828 (26) | 160 (29  221 (40)  57 (10)  118 (21) | 6,243 (25)  11,266 (45)  2,257 (9)  5,389 (21) |
| **Children in the home aged ≤17 years**  Absence of children (2007-2011)  Presence of children (2007-2010)  Recently had kids (2011)  Recent empty nest (2011) | 1,749 (12)  11,003 (72)  366 (2)  2,076 (14) | 856 (12.5)  4,545 (66)  230 (3.4)  1,231 (18) | 81 (14.7)  346 (62.7)  21 (4)  104 (19) | 2,941 (12)  17,536 (70)  673 (3)  3874 (15) |
| **Municipality type**  Metropolitan areas and nearby municipalities  Large cities and surrounding areas  Smaller towns and rural areas | 7,417 (49)  5,537 (36)  2274 (15) | 2,800 (40.4)  2,930 (42.2)  1,207 (17) | 113 (20.5)  261 (47)  178 (32) | 11,036 (44)  9,796 (39)  4,299 (17) |
| **Mental health condition**  None (2007-2011)  Long-term (2007-2010)  Recent (2011) | 12,853 (84)  2,304 (15)  71 (0.5) | 5859 (84)  1053 (15.2)  29 (0.4) | 481 (87)  73 (13)  2 (0.4) | 21,272 (85)  3,769 (15)  114 (0.1) |
| **Physical health condition**  None (2007-2011)  Long-term (2007-2010)  Recent (2011) | 13,740 (90)  1,378 (9.1)  110 (0.7) | 6294 (91)  599 (8.6)  48 (0.7) | 496 (89)  55 (10)  5 (1) | 22,736 (90)  2,236 (9)  183 (0.7) |
| **Cognitive health condition**  None (2007-2011)  Long-term (2007-2010)  Recent (2011) | 15,193 (99.8)  22 (0.1)  13 (0.1) | 6927 (99.8)  12 (0.2)  2 (0.03) | 555 (99.8)  1 (0.2)  0 (0) | 25,096 (99.8)  42 (0.2)  17 (0.1) |

*Note: The stayers and movers are compared based on the same housing situation at baseline using a chi-square test. The table also shows where individuals moved to between 2012 and 2020*

**Supplementary Table** S**6: Descriptive characteristics of stayers and movers who lived in multi-family housing tenant-ownership at baseline, N=17,036**

|  | **Stayers** N=12,016 (%) | **Movers** N=5,020 (%) |
| --- | --- | --- |
| Independent variable | Tenant-owned multi-family housing throughout | |
| **Sex**  Women | 6,405 (53) | 2762 (55) |
| **Education**  Primary  Secondary  Tertiary | 1,664 (14)  5,465 (46)  4,842 (40) | 623 (12)  2,089 (42)  2,283 (46) |
| **Disposable income in quintiles (hundreds of SEK)**  1 (0-1405)  2 (1405-1747)  3 (1747-2314)  4 (2314-3266,4)  5 (3266,4-434310) | 1,372 (11)  1,194 (10)  2,629 (22)  3,243 (27)  3,578 (30) | 592 (12)  406 (8)  950 (19)  1,224 (24)  1,848 (37) |
| **Civil status**  Long-term partnered (2007-2011)  Long-term single (2007-2011)  Recently partnered (2011)  Recently single (2011) | 3,627 (30.1)  5,102 (42.5)  1,461 (12.2)  1,826 (15) | 1,676 (33)  1,640 (33)  548 (11)  1,156 (23) |
| **Children in the home aged ≤17 years**  Absence of children (2007-2011)  Presence of children (2007-2010)  Recently had kids (2011)  Recent empty nest (2011) | 1,153 (10)  8,665 (72)  280 (2)  1,902 (16) | 547 (11)  3,239 (65)  145 (3)  1,063 (21) |
| **Municipality type**  Metropolitan areas and nearby municipalities  Large cities and surrounding areas  Smaller towns and rural areas | 6,705 (56)  3,895 (32)  1,416 (12) | 2,758 (55.1)  1,638 (33)  614 (12.3) |
| **Mental health condition**  None (2007-2011)  Long-term (2007-2010)  Recent (2011) | 10,919 (91)  1074 (9)  23 (0.2) | 4,552 (91)  454 (9)  14 (0.3) |
| **Physical health condition**  None (2007-2011)  Long-term (2007-2010)  Recent (2011) | 11,204 (93)  763 (7)  49 (0.4) | 4,680 (93)  320 (7)  20 (0.4) |
| **Cognitive health condition**  None (2007-2011)  Long-term (2007-2010)  Recent (2011) | 12,007 (99.9)  7 (0.1)  2 (0.02) | 5,013 (99.9)  6 (0.1)  1 (0.02) |

*Note: The stayers and movers are compared based on the same housing situation at baseline using a chi-square test. The table also shows where individuals moved to between 2012 and 2020.*

**Supplementary Table S7 Municipal categories**

| **Metropolitan areas and nearby municipalities (A)** | **Large cities and surrounding areas (B)** | **Smaller towns and rural areas (C)** |
| --- | --- | --- |
| \| Stockholm \| \| --- \| \| Malmö \| \| Göteborg \| \| Upplands Väsby \| \| Vallentuna \| \| Österåker \| \| Värmdö \| \| Järfälla \| \| Ekerö \| \| Huddinge \| \| Botkyrka \| \| Salem \| \| Haninge \| \| Tyresö \| \| Upplands-Bro \| \| Täby \| \| Danderyd \| \| Sollentuna \| \| Nacka \| \| Sundbyberg \| \| Solna \| \| Lidingö \| \| Vaxholm \| \| Sigtuna \| \| Nynäshamn \| \| Håbo \| \| Staffanstorp \| \| Burlöv \| \| Vellinge \| \| Kävlinge \| \| Lomma \| \| Svedala \| \| Skurup \| \| Trelleborg \| \| Kungsbacka \| \| Härryda \| \| Partille \| \| Öckerö \| \| Stenungsund \| \| Ale \| \| Lerum \| \| Bollebygd \| \| Lilla Edet \| \| Mölndal \| \| Kungälv \| \| Alingsås \| | \| Södertälje \| \| --- \| \| Uppsala \| \| Eskilstuna \| \| Linköping \| \| Norrköping \| \| Jönköping \| \| Växjö \| \| Kalmar \| \| Lund \| \| Helsingborg \| \| Kristianstad \| \| Halmstad \| \| Trollhättan \| \| Borås \| \| Karlstad \| \| Örebro \| \| Västerås \| \| Borlänge \| \| Gävle \| \| Sundsvall \| \| Östersund \| \| Umeå \| \| Luleå \| \| Nykvarn \| \| Älvkarleby \| \| Knivsta \| \| Heby \| \| Tierp \| \| Enköping \| \| Gnesta \| \| Strängnäs \| \| Trosa \| \| Kinda \| \| Åtvidaberg \| \| Valdemarsvik \| \| Söderköping \| \| Mjölby \| \| Aneby \| \| Mullsjö \| \| Habo \| \| Vaggeryd \| \| Lessebo \| \| Alvesta \| \| Torsås \| \| Mörbylånga \| \| Sölvesborg \| \| Svalöv \| \| Östra Göinge \| \| Örkelljunga \| \| Bjuv \| \| Sjöbo \| \| Hörby \| \| Bromölla \| \| Perstorp \| \| Klippan \| \| Åstorp \| \| Landskrona \| \| Höganäs \| \| Eslöv \| \| Ängelholm \| \| Laholm \| \| Grästorp \| \| Mark \| \| Svenljunga \| \| Herrljunga \| \| Vänersborg \| \| Kil \| \| Hammarö \| \| Forshaga \| \| Grums \| \| Lekeberg \| \| Hallsberg \| \| Kumla \| \| Nora \| \| Surahammar \| \| Hallstahammar \| \| Sala \| \| Gagnef \| \| Säter \| \| Ockelbo \| \| Timrå \| \| Krokom \| \| Nordmaling \| \| Bjurholm \| \| Robertsfors \| \| Vännäs \| \| Östhammar \| \| Finspång \| \| Motala \| \| Nässjö \| \| Uppvidinge \| \| Tingsryd \| \| Nybro \| \| Hässleholm \| \| Hylte \| \| Tranemo \| \| Uddevalla \| \| Ulricehamn \| \| Munkfors \| \| Kristinehamn \| \| Laxå \| \| Askersund \| \| Lindesberg \| \| Köping \| \| Sandviken \| \| Bräcke \| \| Berg \| \| Vindeln \| \| Älvsbyn \| \| Boden \| | \| Norrtälje \| \| --- \| \| Nyköping \| \| Katrineholm \| \| Värnamo \| \| Ljungby \| \| Oskarshamn \| \| Västervik \| \| Gotland \| \| Karlskrona \| \| Karlshamn \| \| Ystad \| \| Falkenberg \| \| Varberg \| \| Mariestad \| \| Lidköping \| \| Skövde \| \| Falköping \| \| Karlskoga \| \| Falun \| \| Avesta \| \| Ludvika \| \| Hudiksvall \| \| Härnösand \| \| Örnsköldsvik \| \| Skellefteå \| \| Piteå \| \| Kiruna \| \| Vingåker \| \| Oxelösund \| \| Flen \| \| Ödeshög \| \| Ydre \| \| Boxholm \| \| Vadstena \| \| Gnosjö \| \| Sävsjö \| \| Eksjö \| \| Älmhult \| \| Markaryd \| \| Högsby \| \| Hultsfred \| \| Mönsterås \| \| Emmaboda \| \| Olofström \| \| Ronneby \| \| Höör \| \| Tomelilla \| \| Osby \| \| Tjörn \| \| Orust \| \| Munkedal \| \| Dals-Ed \| \| Färgelanda \| \| Vårgårda \| \| Essunga \| \| Karlsborg \| \| Gullspång \| \| Mellerud \| \| Vara \| \| Götene \| \| Tibro \| \| Töreboda \| \| Åmål \| \| Skara \| \| Hjo \| \| Tidaholm \| \| Storfors \| \| Degerfors \| \| Ljusnarsberg \| \| Skinnskatteberg \| \| Kungsör \| \| Norberg \| \| Fagersta \| \| Arboga \| \| Smedjebacken \| \| Hedemora \| \| Hofors \| \| Nordanstig \| \| Gislaved \| \| Vetlanda \| \| Tranås \| \| Vimmerby \| \| Bengtsfors \| \| Lysekil \| \| Torsby \| \| Sunne \| \| Filipstad \| \| Hagfors \| \| Arvika \| \| Säffle \| \| Hällefors \| \| Vansbro \| \| Ovanåker \| \| Ljusdal \| \| Söderhamn \| \| Bollnäs \| \| Ånge \| \| Kramfors \| \| Sollefteå \| \| Ragunda \| \| Strömsund \| \| Norsjö \| \| Malå \| \| Dorotea \| \| Vilhelmina \| \| Åsele \| \| Lycksele \| \| Arvidsjaur \| \| Överkalix \| \| Kalix \| \| Övertorneå \| \| Pajala \| \| Haparanda \| \| Borgholm \| \| Båstad \| \| Simrishamn \| \| Sotenäs \| \| Tanum \| \| Strömstad \| \| Eda \| \| Årjäng \| \| Malung-Sälen \| \| Leksand \| \| Rättvik \| \| Orsa \| \| Älvdalen \| \| Mora \| \| Åre \| \| Härjedalen \| \| Storuman \| \| Sorsele \| \| Arjeplog \| \| Jokkmokk \| \| Gällivare \| |

*
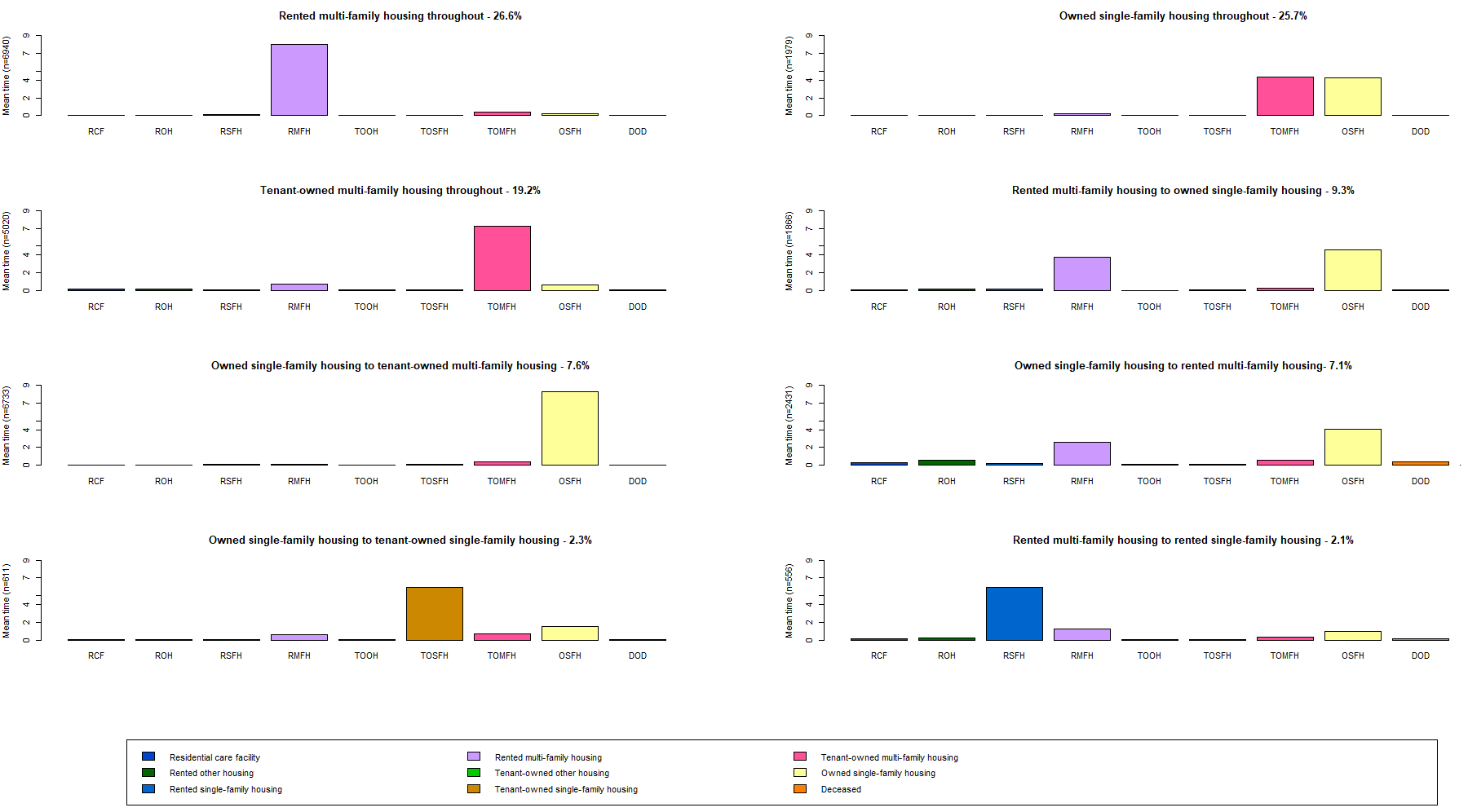
*

*Supplementary Figure S8: Mean time spent in each housing state during the study period*


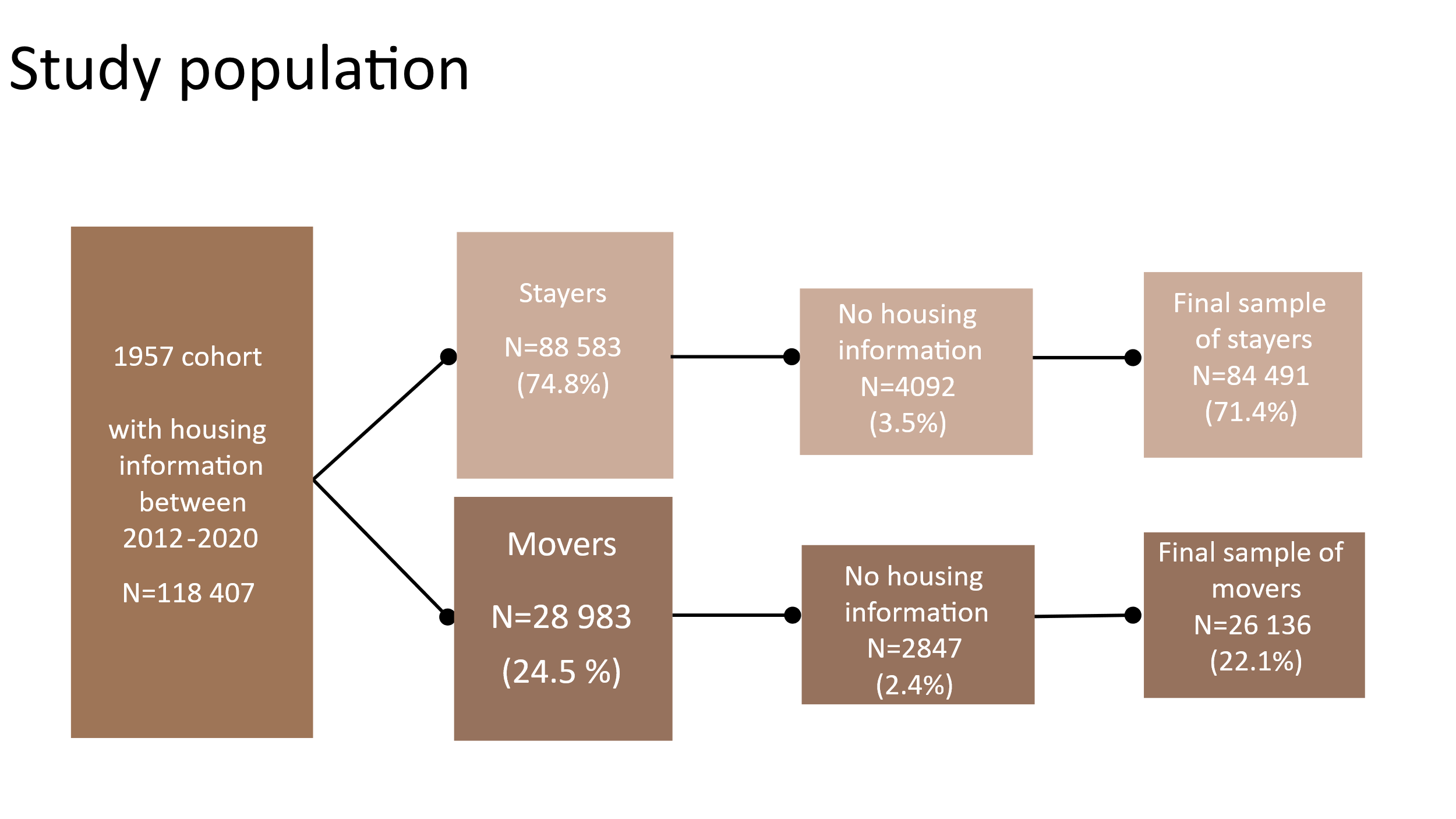


Supplementary Figure S9: Flow chart of excluded individuals
